# Alterations in plasma soluble vascular endothelial growth factor receptor-1 (sFlt-1) concentrations during coronary artery bypass graft surgery: relationships with post-operative complications

**DOI:** 10.1186/1749-8090-3-16

**Published:** 2008-04-18

**Authors:** Yves Denizot, Alexandre Leguyader, Elisabeth Cornu, Marc Laskar, Isabelle Orsel, Christelle Vincent, Nathalie Nathan

**Affiliations:** 1 UMR CNRS 6101, Centre National de la Recherche Scientifique, Université de Limoges, France; 2 Service de Chirurgie Thoracique et Cardiovasculaire, CHU Dupuytren, Limoges, France; 3 Service d'Anesthésie Réanimation Chirurgicale, CHU Dupuytren, Limoges, France

## Abstract

**Background:**

Plasma concentrations of sFlt-1, the soluble form of the vascular endothelial growth factor receptor (VEGF), markedly increase during coronary artery bypass graft (CABG) surgery with extracorporeal circulation (ECC). We investigated if plasma sFlt-1 values might be related to the occurrence of surgical complications after CABG.

**Methods:**

Plasma samples were collected from the radial artery catheter before vascular cannulation and after opening the chest, at the end of ECC just before clamp release, after cross release, after weaning from ECC, at the 6^th ^and 24^th ^post-operative hour. Thirty one patients were investigated. The presence of cardiovascular, haematological and respiratory dysfunctions was prospectively assessed. Plasma sFlt-1 levels were measured with commercially ELISA kits.

**Results:**

Among the 31 investigated patients, 15 had uneventful surgery. Patients with and without complications had similar pre-operative plasma sFlt-1 levels. Lowered plasma sFlt-1 levels were observed at the end of ECC in patients with haematological (p = 0.001, ANOVA) or cardiovascular (p = 0.006) impairments, but not with respiratory ones (p = 0.053), as compared to patients with uneventful surgery.

**Conclusion:**

These results identify an association between specific post-CABG complication and the lower release of sFlt-1 during ECC. sFlt-1-induced VEGF neutralisation might, thus, be beneficial to reduce the development of post-operative adverse effects after CABG.

## Introduction

Coronary artery bypass graft (CABG) surgery with extracorporeal circulation (ECC) is associated with an inflammatory response [1,2]. Alterations of lipidic, cytokine, and colony stimulating factor (CSF) networks observed during and after CABG surgery might be involved in the post-CABG multiple organ failure syndrome [3-7]. The angiogenic network was also affected during and after CABG [8-10]. Among angiogenic growth factors, vascular endothelial growth factor (VEGF) fulfils a central role in the formation and function of blood vessels and during vascular healing in response, for example, to vascular trauma induced by mechanical disruption [11,12]. In vivo VEGF induces angiogenesis as well as permeabilisation of blood vessels and play central role in the regulation of vasculogenesis [12]. VEGF acts through two membrane receptors, Flt-1 (VEGFR-1) and KDR (VEGFR-2) which belong to the receptor tyrosine kinase superfamily. Soluble forms of Flt-1 (sFlt-1) and KDR (sKDR) are found in human plasma [12]. Studies highlighted that sFlt-1 is capable of sequestering VEGF and preventing signal transduction [12]. Moreover, sFlt-1 can act as an endogenous VEGF inhibitor not only by sequestering VEGF but also by binding and inactivating membrane-bound Flt-1 and KDR receptors through a mechanism involving receptor homodimerisation or heterodimerisation [13]. Recently we reported the release of sFlt-1 (but not sKDR) during uneventful CABG surgery and hypothesized that sFlt-1 by neutralizing VEGF and/or by inactivating membrane-bound Flt-1 and KDR receptors, might play a role in the occurrence of post-CABG complications [14]. In order to test this hypothesis we investigated whether the plasma levels of sFlt-1 was related to complication after CABG.

## Patients and methods

### Study population

According to the principles outlined in the Declaration of Helsinki and in agreement with the ethic committee of our University Hospital, a total of 31 patients were included in a study investigating cytokine and lipid mediator blood concentrations during CABG from January 1995 and December 1996. Patients were selected on a consecutive manner according to the availability of investigators. Only patients with a preoperative ejection fraction >40% were included. All patients consented in an informed manner that samples might be use later in order to investigate the involvement of new factors. After completion of the study, approximately 200 plasma samples drawn before, during and after CABG were analysed for various lipid mediators (platelet-activating factor, 6-keto prostaglandin F_1α_, prostaglandin E_2_, thromboxane B_2_, leukotriene B_4 _and C_4_), cytokines (interleukin 4, 6, 8, 10 and 13, tumor necrosis factor α, leukaemia inhibitory factor), growth factors (macrophage-CSF, granulocyte-CSF, granulocyte-macrophage-CSF), and angiogenic factors (VEGF, basic fibroblast growth factor, epidermal growth factor and transforming growth factor β1). Manuscripts on the involvement of these compounds during CABG have been published separately [3-7]. At the time of the present study, remaining plasma samples from 31 patients were available for analysis of sFlt-1 levels. All plasma samples were stored at -80°C until used. All samples undergo the same number of freeze-thaw cycles (not more than 3). All samples (from T0 to T5) were tested the same day.

All patients had a preoperative ejection fraction above 40%. Anaesthesia was induced and maintained with titrated doses of fentanyl and flunitrazepam. Muscular relaxation was achieved with pancuronium (0.1 mg/kg). Membrane oxygenators were used. All patients received 300 U/kg of heparin just before vascular cannulation. Incremental 100 U boluses of heparin were added each hour during the procedure to maintain an activated clotting time greater than 600 seconds. At the end of ECC protamine was administered in a 2:3 ratio with heparin. The blood was harvested from the surgical field and from the cell saver at the end of ECC and reinfused to all patients. All patients received high doses of aprotinin and no patients received corticoids. The following criteria were used for weaning patients from ventilator. PaO_2_:FiO_2 _ratio > 300, hemodynamic stability without vasoactive drugs, complete rewarming of patients, no bleeding complications.

Plasma samples were collected from the radial artery catheter before vascular cannulation and after opening the chest (T_0_), at the end of ECC just before (T_1_) and after cross clamp release (T_2_), after weaning from ECC (T_3_), at the 6^th ^(T_4_) and 24^th ^post-operative hour (T_5_). The presence of complications was prospectively assessed. Cardiovascular dysfunction was determined at T_3_, T_4 _and T_5 _according to the need of inotropic drugs to obtain a systolic arterial pressure (SAP) above 90 mm Hg. Myocardial infarction and dysrhythmias were recorded. Pulmonary artery occlusion pressure (PAOP), control veinus pressure (CVP) and cardiac (and subsequent derived measures) output were monitored by a swan ganz catheter. At the time of the protocol systems monitoring blood volume by analysis of arterial curve (such as Picco or Vigileo^®^) were not available. Hematological complications were ensured at T_3_, T_4 _and T_5 _by the presence of one of the following biological disorders: activated partial thromboplastin time values > 1.5 greater than control, fibrinogen level < 1 g/L, factor V level < 30%, platelet count < 70,000 elements/mm^3 ^or clinical bleeding (blood loss greater than 100 mL/h). At T_2 _and T_3_, the Murray Lung Injury Score (without measurements of compliances) was use to ensure the presence of respiratory complications [15].

### Measurements of plasma sFlt-1 levels

Plasma sFlt-1 levels were measured with commercially ELISA kits (R&D Systems, Abingdon, UK). Biological results were expressed by pg/mg of protein measured at the simultaneous times. The sensitivity of the assays enables the detection of levels as low as 20 pg/ml of sFlt-1. The standard curve was linear, from 30 to 1000 pg/ml, and plasma samples above this range was diluted by 1/10. Results are expressed as mean ± SEM. The proteinemia was determined by the BCA Protein Assay Reagent (Pierce, Rockford, IL). No significant differences were found for protein contents after 1 and 3 freeze-thaw cycles (data not shown).

### Statistical analyses

Statistical analysis was performed by using analysis of variance (ANOVA) for repeated measures (including the time and the type of complication as covariables). Results were considered statistically significant when *P *< 0.05.

## Results and discussion

Among the 31 investigated patients, 15 had uneventful surgery and 16 exhibited adverse outcomes. All these adverse outcomes were summarized in Table 1. Four patients had transient vital complications (including 1 with no evident sign of cardiovascular, haematological and respiratory impairments; 1 with cardiovascular impairments; 1 with cardiovascular and respiratory impairments; 1 with cardiovascular, haematological and respiratory impairments). These 4 patients were weaned from the ventilator at the 24^th ^post-operative hour. Twelve patients had persistent vital complications (3 with respiratory impairments, 3 with cardiovascular and respiratory impairments; 1 with cardiovascular and haematological impairments; 3 with hematological and respiratory impairments; 2 with cardiovascular, respiratory and hematological impairments). Among them, three died postoperatively at day 3, 6 and 14, respectively. No significant difference could be documented between the two groups on several parameters including age, gender ratio, weight, body surface area, ejection fraction, preoperative left ventricular end diastolic pressure, number of grafts, ECC duration, heparin total dose, protamine total dose, preoperative blood infusion and blood reinfused by the cell saver (data not shown).

**Table 1:** Type of impairments in complicated patients

Type of impairments	Number of patients (n = 16)
Cardiovascular	1
Haematological	0
Respiratory	3
Cardiovascular and haematological	1
Cardovascular and respiratory	4
Haematological and respiratory	3
Cardiovascular, haematological and respiratory	3
No evident	1

**Figure 1:**
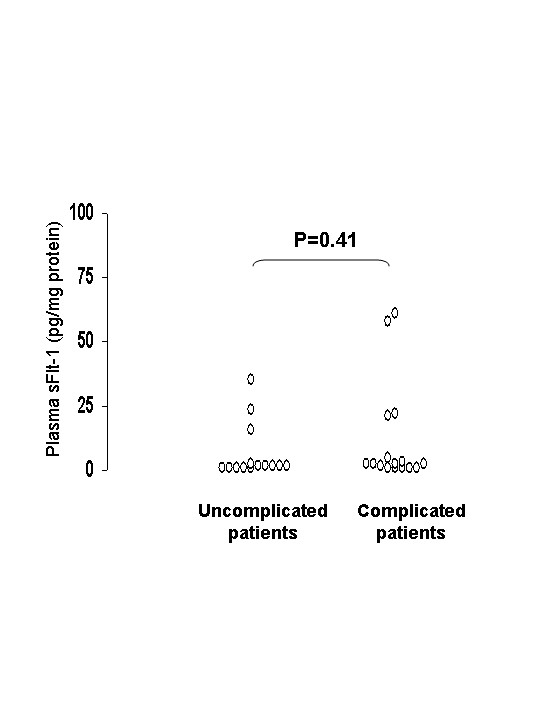
**Plasma sFlt1 before cardiopulmonary bypass graft surgery**. Plasma sFlt-1 values were investigated before surgery in uncomplicated (n = 15) and complicated (n = 16) patients. Values are expressed in pg per mg protein. Mann-Whitney U-test was used to compare groups.

A decrease of serum/plasma protein contents was observed during and after CABG surgery [16]. To avoid the influence of haemodilution during bypass, sFlt-1 levels were expressed as ng/mg of total protein contents measured at the simultaneous times. Plasma sFlt-1 values of patients before CABG are shown in Figure 1. The mean sFlt-1 plasma concentration was 5.9 pg/mg protein (range 0.6–35.0) in the uneventful group and 11.5 (range 0.7–61.0) in the complicated group. This difference was not statistically significant (p = 0.41, Mann-Whitney U-test). Raw data of patients (without proteinemia correction) were 301.6 pg/ml (range 30–1680) in the uneventful group and 475.0 pg/ml (range 30–2660) in the complicated group. Thus, pre-operative plasma sFlt-1 could not be used as a prognostic marker of post-operative complication after CABG surgery. The values plasma concentrations reported here are similar like from those detected in plasma from serum volunteers [17], showing that long freezing and freeze-thaw cycles had any effect on sFlt-1 levels. Moreover since similar pre-operative plasma VEGF levels were found in these patients [7] abnormal indices of angiogenesis were not evident between our two groups of patients.

**Figure 2:**
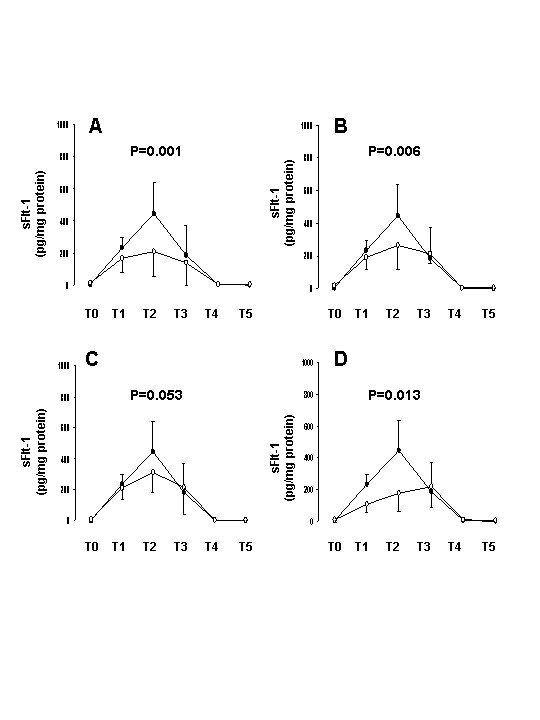
**Comparison of plasma sFlt-1 levels for complicated and uncomplicated patients during the course of CABG**. Plasma sFlt-1 values are expressed in pg per mg protein. T_0_: before vascular cannulation and after opening the chest; T_1_: during extracorporeal circulation (ECC); T_2_: at the end of ECC before cross clamp release; T_3_: after cross clamp release; T_4_: the 6^th ^post-operative hour; T_5_: the 24^th ^post-operative hour. A: sFlt-1 levels in 15 uncomplicated patients (solid squares) and 7 patients with hematological impairments (open squares). B: sFlt-1 levels in 15 uncomplicated patients (solid squares) and 9 patients with cardiovascular impairments (open squares). C: sFlt-1 levels in 15 uncomplicated patients (solid squares) and 13 patients with respiratory impairments (open squares). D: sFlt-1 levels in 15 uncomplicated patients (solid squares) and 3 patients who died postoperatively (open squares). Results were expressed as means ± SD Statistical differences were made using ANOVA test.

When sFlt-1 was evaluated during the course of CABG surgery significant differences were found between complicated and uneventful patients. Thus, lower circulating sFlt-1 levels were observed in patients with hematological (p = 0.001, ANOVA test) and cardiovascular (p = 0.006) impairments (Figure 2A and 2B, respectively). In contrast sFlt-1 values were not significantly (p = 0.053) different in patients with respiratory impairments as compared with uneventful patients (Fig 2C). Finally lower sFlt-1 levels (p = 0.013) were also evidenced for the 3 patients who died postoperatively (Figure 2D). This study confirms in a set of complicated patients the release of sFlt-1 during CABG with ECC; sFlt-1 values of uneventful patients being previously reported separately [14]. In this study all patients received aprotinin. The use of aprotinin in cardiac surgery is currently the subject of much debate since aprotinin might have modified the inflammatory response of our patients [18]. However all patients (both uneventful and complicated) received aprotinin. Thus, it is not the use of aprotinin by itself that reduced sFlt-1 release in complicated patients. In this study we investigated CABG surgery with ECC. In the last few years, off-pump coronary artery bypass grafting has gained widespread attention as an alternative technique to conventional on-pump coronary artery bypass grafting. However the available literature does not permit definitive conclusions to be made on the advantages of off-pump surgery with respect to the systemic inflammatory response [19]. Moreover mortality, stroke, myocardial infarction and renal failure were not improved [20]. Clearly investigation of sFlt-1 and VEGF release during off-pump cardiac surgery would be of interest to ensure the involvement of angiogenic/anti-angionic pathways during this type of cardiac surgery.

Data have demonstrated elevated VEGF levels following CABG surgery [8,9], suggesting that VEGF is a component of the inflammatory network after open heart surgery. In a previous study we reported that plasma VEGF levels were higher in patients with cardiovascular and hematological impairments as compared with patients with uneventful surgery [7]. The main finding of the present study is that patients who developed cardiovascular or haematological dysfunctions after CABG surgery had lower sFlt-1 levels as compared with uncomplicated ones. Results suggest that sFlt-1 production might play a protective role in the occurrence of post-CABG complications. These results are in agreement with data reporting decreased levels of sFlt-1 and elevated levels of VEGF in patients with coronary artery disease or myocardial infarction [21,22]. In culture sFlt-1 has been shown to block VEGF effects [12,13]. Animal models have shown the interest of sFlt-1 infusion to improve cardiac function in a mouse model of sepsis [23] and to reduce disease severity in experimental inflammatory diseases [24,25].

Two possible mechanisms may be suggested to explain the role of sFlt-1 in cardiac surgery. It is possible that sFlt-1 might act by sequestering VEGF and, thus, preventing signal transduction [12]. It is also possible that sFlt-1 can act as an endogenous VEGF inhibitor by binding and inactivating membrane-bound Flt-1 and KDR receptors [13]. We favour the second hypothesis since VEGF levels picked at the 6^th ^and 24^th ^post-operative hours [7] while sFlt-1 levels picked during ECC. These kinetics of sFlt-1 and VEGF release might indicate a similar sFlt-1 mechanism for the diverse outcomes observed (cardiovascular and haematological impairments).

## Conclusion

Results of the current study strengthened the hypothesis of a pathophysiological role for VEGF in mediating post-operative complications and that VEGF neutralisation in vivo appears beneficial to reduce the development of these post-operative adverse effects. However the present work is an observational study that cannot, as such, establish a link of causality between sFlt-1 levels and adverse outcomes after CABG. Only a clinical study investigating the influence of sFlt-1 infusion on post-operative adverse effects after CABG might establish such a link.

## Abbreviations

CABG: coronary artery bypass graft, ECC: extracorporeal circulation, VEGF: vascular endothelial growth factor

## Competing interests

The authors declare that they have no competing interests.

## Authors' contributions

AL, EC, ML, IO and NN were the surgical and anaesthesia teams and carried out the preoperative clinical and analytical data collection, carried out blood samples and actively participate to the study design, data interpretation and manuscript writing. YD and CV carried out the determination of sFlt-1 and actively participate to manuscript writing. All authors read and approved the final manuscript.
